# Inhibition of projections from the basolateral amygdala to the entorhinal cortex disrupts the acquisition of contextual fear

**DOI:** 10.3389/fnbeh.2014.00129

**Published:** 2014-05-06

**Authors:** Dennis R. Sparta, Jim Smithuis, Alice M. Stamatakis, Joshua H. Jennings, Pranish A. Kantak, Randall L. Ung, Garret D. Stuber

**Affiliations:** ^1^Departments of Psychiatry and Cell Biology and Physiology, UNC Neuroscience Center, University of North Carolina at Chapel HillChapel Hill, NC, USA; ^2^Bowles Center for Alcohol Studies, University of North Carolina at Chapel HillChapel Hill, NC, USA; ^3^Curriculum in Neurobiology, University of North Carolina at Chapel HillChapel Hill, NC, USA

**Keywords:** optogenetics, hippocampus, channelrhodopsin-2, halorhodopsin, fear conditioning, amygdala, glutamate

## Abstract

The development of excessive fear and/or stress responses to environmental cues such as contexts associated with a traumatic event is a hallmark of post-traumatic stress disorder (PTSD). The basolateral amygdala (BLA) has been implicated as a key structure mediating contextual fear conditioning. In addition, the hippocampus has an integral role in the encoding and processing of contexts associated with strong, salient stimuli such as fear. Given that both the BLA and hippocampus play an important role in the regulation of contextual fear conditioning, examining the functional connectivity between these two structures may elucidate a role for this pathway in the development of PTSD. Here, we used optogenetic strategies to demonstrate that the BLA sends a strong glutamatergic projection to the hippocampal formation through the entorhinal cortex (EC). Next, we photoinhibited glutamatergic fibers from the BLA terminating in the EC during the acquisition or expression of contextual fear conditioning. In mice that received optical inhibition of the BLA-to-EC pathway during the acquisition session, we observed a significant decrease in freezing behavior in a context re-exposure session. In contrast, we observed no differences in freezing behavior in mice that were only photoinhibited during the context re-exposure session. These data demonstrate an important role for the BLA-to-EC glutamatergic pathway in the acquisition of contextual fear conditioning.

## Introduction

In classical fear conditioning, an aversive stimulus (unconditioned stimulus (US)) is coupled to a neutral stimulus (conditioned stimulus (CS)). After successful pairings, the CS by itself will elicit aversive responses such as freezing, increases in arterial blood flow, and enhanced skin conductance (Blanchard and Blanchard, 1969; Kapp et al., 1979; Fanselow, 1980). Importantly, contextual information associated with the aversive stimuli has been shown to be involved in the expression of conditioned fear responses (Bouton and King, 1983; Harris et al., 2000; Bouton, 2002). Deficits in contextual fear conditioning can lead to the development of anxiety disorders, such as post-traumatic stress disorder (PTSD; Maren et al., 2013). Approximately 10% of the population that experience a traumatic event will develop PTSD (de Vries and Olff, 2009; Koch et al., 2014). PTSD is characterized by excessive fear and/or stress responses to cues associated with the traumatic event. Clinical studies show that PTSD patients have alterations in the processing of contextual information associated with aversive stimuli (Rougemont-Bucking et al., 2011). Although brain regions such as the prefrontal cortex, extended amygdala and hippocampus have been implicated in the development of PTSD, the functional connectivity between these structures remains elusive (Liberzon and Sripada, 2008).

A large body of evidence suggests that the basolateral amygdala (BLA) is essential for the formation of emotional memories as well as fear conditioning (Davis, 1992, 1997; Phillips and LeDoux, 1992; Lavond et al., 1993; Goosens and Maren, 2003; Zimmerman and Maren, 2010). Pharmacological blockade as well as lesions of the BLA can block both cue-and contextual fear conditioning (Blanchard and Blanchard, 1972; Gentile et al., 1986; Hitchcock and Davis, 1986; Goosens and Maren, 2001). While these studies indicate a key role for the BLA in both cue-and contextual fear conditioning, evidence suggest that these two distinct types of conditioning require different neuronal circuits within the BLA (Hall et al., 2001). Due to its role in spatial and contextual processing, the hippocampus remains a potential downstream target of BLA neurons involved in contextual fear (Winocur and Olds, 1978; Nadel, 1987; Kim et al., 1993; Maren and Fanselow, 1997). Although the BLA sends a weak projection to the CA1, it’s main projection to the hippocampal formation is through the entorhinal cortex (EC; Pitkänen et al., 2000). Several studies indicate that the EC is integral for the processing of contextual information and relaying that information from the BLA to the hippocampal formation (Ueki et al., 1994; Maren and Fanselow, 1997; Majchrzak et al., 2006).

There are several studies that have examined the roles of both the BLA and hippocampus in regards to fear conditioning. Lesions of the BLA lead to decreased freezing behavior in both cued- and contextual fear conditioning, whereas lesions of the hippocampus lead to attenuated freezing behavior in only contextual fear conditioning (Phillips and LeDoux, 1992). At the gross anatomical level, these studies demonstrate that both structures play an important role in aversive learning, but whether direct functional connectivity from the BLA to the EC regulates acquisition or expression of contextual fear is unknown. Therefore, in the present study, we used an inhibitory optogenetic approach to examine the necessity of the BLA-EC pathway during the acquisition and expression of contextual fear.

## Materials and methods

### Experimental subjects

Adult (≥22 g) male C57BL/6J mice (Jackson Labs, Bar Harbor, ME) were used as subjects. All mice were group housed with littermates prior to surgical procedures (see below), after which all subjects were singly housed. Mice were maintained on a 12:12 light cycle (lights on at 19:00) and given *ad libitum* food and water access prior to behavioral training. All experiments were conducted in accordance with guidelines of the University of North Carolina Animal Care and Use Committee.

### Stereotaxic recombinant adeno-associated virus injection

Mice were anesthetized with ketamine (100 mg/kg, i.p.) and xylazine (10 mg/kg, i.p) and placed in a stereotaxic frame (Kopf Instruments). Microinjection needles were inserted above the BLA (coordinates from Bregma: −1.6 AP, ±3.3 ML, −4.45 DV). For all slice physiology experiments, each BLA was injected with 0.5 μl of purified and concentrated adeno-associated virus (AAV; ∼10^12^ infectious units/mL, serotype 5) to express channelrhodopsin-2 fused to eYFP under control of the CamKIIα promoter (AAV-CaMKIIα-ChR2(H134R)-eYFP). For all behavior experiments, each BLA was injected with 0.5 μl of purified and concentrated AAV (∼10^12^ infectious units/mL, serotype 5) to express halorhodopsin version 3.0 fused to eYFP under control of the CamKIIα promoter (AAV-CaMKIIα-NpHR3.0-eYFP; Gradinaru et al., 2010) or only eYFP under control of the CamKIIα promoter (AAV-CamKIIα-eYFP) as a control group, over 5 min followed by 5 min to allow diffusion of viral particles away from the injection site. For all *in vivo* behavioral experiments, optical fibers were implanted above the EC bilaterally (coordinates from Bregma: −2.9 AP, ±3.2 ML, −4.3 DV). Optical fibers were constructed in house using the following protocol (Sparta et al., 2011). Optical fibers had an inner core diameter of 200 μm. Light intensity was set to 10 mW per hemisphere, in order to illuminate ∼1 mm^3^ of tissue *in vivo*. Mice were allowed at least 2 weeks to recover prior to behavioral training.

### Patch-clamp electrophysiology

Brain slice preparation and general methods for patch-clamp electrophysiology were conducted as previously described (Stamatakis and Stuber, 2012; van Zessen et al., 2012; Jennings et al., 2013). To examine BLA post-synaptic currents evoked by optical stimulation of glutamatergic neurons, 200 μm coronal slices containing the EC were prepared from mice expressing ChR2-eYFP in BLA glutamatergic terminals. For whole-cell voltage clamp recordings, (EPSCs) from EC neurons, electrodes (2–4 MΩ electrode resistance) contained in mM: 117 cesium methanesulfonate, 20 HEPES, 0.4 EGTA, 2.8 NaCl, 5 TEA, 2 Mg-ATP, 0.2 Na-GTP (pH 7.2–7.4), 275–285 mOsm. Photostimulation (5 ms pulses of 1–2 mW, 473 nm light delivery via LED through a 40 × microscope objective) was used to stimulate BLA glutamatergic terminals expressing ChR2-eYFP. For paired pulse experiments, we pulsed 5 ms of light separated by 50 ms. All cells were held at −70 mV.

### Contextual fear conditioning

#### Behavioral apparatus

Following surgery, all mice underwent behavioral testing in a standard fear-conditioning chamber (CS; Med Associates Inc.) The chamber (21.6 × 17.8 × 12.7 cm) was constructed of aluminum (side walls) and polycarbonate (front, back, and top) and was placed in a sound attenuating cabinet. The floor of the chamber consisted of stainless steel rods that were connected to an aversive shock generator for the delivery of a footshock. Additionally, each box had a laser (532 nm) that attached to a commutator (Doric Lenses) in order to transmit green light to inhibit glutamate fibers from the BLA to the EC.

#### Fear conditioning procedure

All mice underwent 3 days of behavioral testing. On Day 1, all mice would receive 4 foot shocks (US; 0.6 mA/0.5 s) each minute during a 5 min acquisition session (20 foot shocks total). A subset of mice (8 *CamKIIα*^BLA-EC^::NpHR3.0; 8 *CamKIIα*^BLA-EC^::eYFP) received laser stimulation during this entire acquisition session (5 min constant). On Day 2, these mice were re-exposed to the context, but did not receive any shocks. A separate cohort of mice (9 *CamKIIα*^BLA-EC^::NpHR3.0; 8 *CamKIIα*^BLA-EC^::eYFP) received laser stimulation during the entire expression session (5 min constant). One week later all mice were re-exposed to the same context in the absence of shock for a second expression session (Day 3; see Figure 2B for behavior timing schematic). To assess fear-like behavior, we analyzed freezing using Ethovision 8.0 (Noldus Information Tech). Freezing was defined by the suppression of all visible movements except those needed for respiration and scored by Ethovision, using a maximum movement threshold for freezing of 3.5% pixel change per sample (10 samples/s).

**Figure 1:**
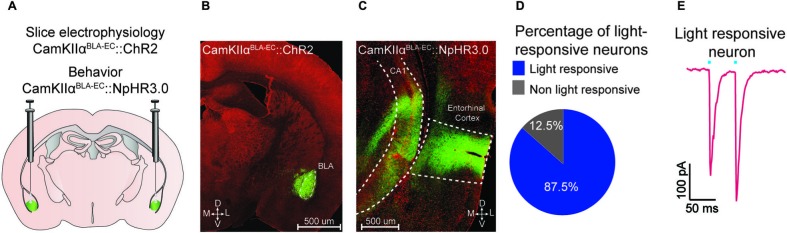
**Optogenetic analysis of the BLA-EC pathway. (A)** Schematic depicting viral delivery method of AAV5-DIO-ChR2-eYFP, AAV5-DIO-NpHR3.0-eYFP, and AAV5-DIO-eYFP into the BLA of C57Bl/6J mice. **(B)** A representative coronal section of the BLA with expression of ChR2-eYFP (green) in pyramidal neurons within the BLA. Red, counterstaining with 640 nm Neurotrace to label all neuronal cell bodies. (D = dorsal; V = ventral; M = medial; L = lateral; scale bar = 500 μm). **(C)** A representative coronal section of the EC with expression of ChR2-eYFP (green) in glutamatergic fibers originating from the BLA (scale bar = 200 μm). **(D)** Percentage of light responsive vs. non-light responsive neurons in the EC following photostimulation of BLA glutamatergic fibers. **(E)** Example trace of a light responsive neuron in the EC. All values for all figures represent mean ± s.e.m.

**Figure 2:**
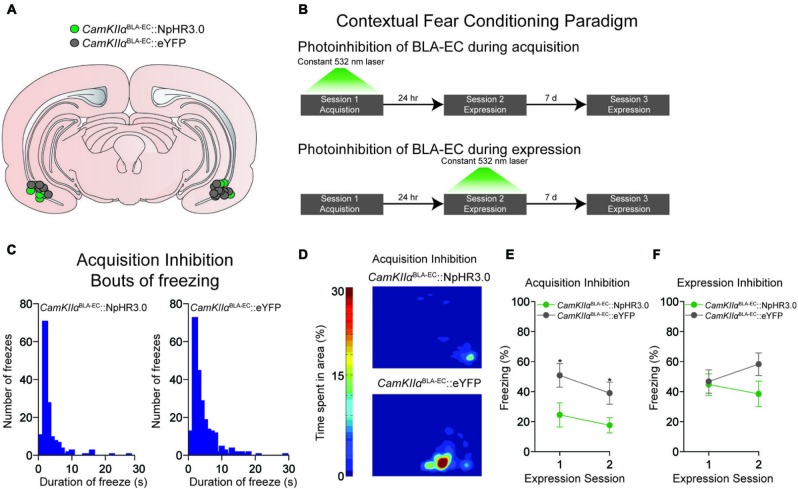
**Photoinhibition of the glutamatergic pathway from the BLA to the EC during contextual fear conditioning. (A)** Schematic diagram showing optical fiber placement for *CamKIIα*^BLA-EC^::NpHR3.0 (*n* = 17 mice) and *CamKIIα*^BLA-EC^::eYFP (*n* = 16 mice) in the EC for all behavioral experiments (D = dorsal; V = ventral; M = medial; L = lateral). **(B)** Schematic illustrating the different contextual dear conditioning paradigms employed. **(C)** Histogram depicting the number of freezes during the first expression session in the *CamKIIα*^BLA-EC^::NpHR3.0 group (left) and *CamKIIα*^BLA-EC^::eYFP (right) after laser inhibition during the acquisition session. Length of freezes were cut off at 30 s, although there were a few bouts (1 in the *CamKIIα*^BLA-EC^::NpHR3.0 group; 3 in the *CamKIIα*^BLA-EC^::eYFP group) that lasted longer than our 30 s cutoff time epoch. **(D)** Representative heat maps displaying average time spent in the context during the first expression session after laser inhibition during the acquisition session from *CamKIIα*^BLA-EC^::NpHR3.0 (top) and *CamKIIα*^BLA-EC^::eYFP (bottom) mice. **(E)** Average freezing (%) of *CamKIIα*^BLA-EC^::NpHR3.0 (*n* = 8 mice) and *CamKIIα*^BLA-EC^::eYFP (*n* = 8 mice) during the two expression sessions after laser stimulation during the acquisition session. *CamKIIα*^BLA-EC^::NpHR3.0 mice spent significantly less time frozen during both expression sessions compared to *CamKIIα*^BLA-EC^::eYFP. **(F)** Average freezing (%) of *CamKIIα*^BLA-EC^::NpHR3.0 (*n* = 9 mice) and *CamKIIα*^BLA-EC^::eYFP (*n* = 8 mice) during two expression sessions after laser stimulation during the 1st expression session, showing no differences between the groups. All values for all figures represent mean ± s.e.m. * *P* < 0.05.

### Data analysis

All values are presented as means ± SEM. Statistical analysis was assessed using repeated measures analysis of variance (ANOVA). When statistical significance was achieved, *post-hoc* tests (independent and paired *t*-tests) were conducted to compare group means, using α = 0.05 and calculated with SPSS (IBM). No significance (ns) defined as *P* > 0.05, and significance defined as * *P* < 0.05, ** *P* < 0.001.

## Results

### Optogenetic targeting of glutamatergic terminals in the EC from the BLA

We targeted the expression of ChR2-eYFP, NpHR3.0-eYFP or eYFP to glutamatergic neurons of the BLA by injecting AAV constructs under control of the CamKIIα promoter into the BLA of C57Bl/6J mice (Figure 1A). Confocal microscopy revealed robust eYFP expression throughout the BLA (Figure 1B) and in BLA projection fields that innervate the EC (Figure 1C). To examine the functional connectivity between glutamatergic BLA neurons and EC neurons, we performed patch clamp electrophysiology on EC neurons. We found that photostimulation of BLA glutamatergic fibers produced excitatory post-synaptic currents onto 87.5% (49/56 neurons) of EC neurons (Figure 1D). Additionally, we observed no significant difference in paired pulse ratio (*p*1 = −192.7 pA; *p*2 = −247.2 pA; *n* = 13 EC neurons) after photostimulation of ChR2-transduced BLA terminals (Figure 1E).

### Photoinhibition of *CamKII*α^BLA-EC^ pathway during acquisition disrupts the expression of contextual fear

Next, we determined whether constant photoinhibition of the *CamKIIα*^BLA-EC^ pathway during acquisition of contextual fear conditioning altered the subsequent expression of freezing behavior 24 h later. Mice were injected with an AAV construct coding either NpHR3.0-eYFP or eYFP under control of the CamKIIα promoter into the BLA, as well as implanted with optical fibers bilaterally above the EC (Figure 2A). We found that constant optogenetic inhibition of BLA-EC projections during the acquisition session resulted in significant attenuation of freezing when mice were returned to the contextual fear environment 24 h later compared to controls (repeated measures ANOVA NpHR3.0 vs. eYFP: *F*_1,14_ = 6.060; *P* = 0.027; independent *t* during 1st expression test: *t*_14_ = −2.344; *P* = 0.034) (Figures 2B–E). This corresponded to an increase in both bouts and duration of freezing events in the *CamKIIα*^BLA-EC^::eYFP group compared to *CamKIIα*^BLA-EC^::NpHR3.0 group (Figures 2C–D). Interestingly, the ability of BLA-EC inhibition during contextual fear acquisition to reduce fear expression was long lasting, as freezing was significantly attenuated when mice were re-tested 7 days following the first expression session (independent *t* during 2nd expression test: *t*_14_ = −2.393; *P* = 0.031) (Figure 2E). Taken together, these data demonstrate that suppression of BLA-EC transmission disrupts the acquisition of contextual fear.

In a final set of behavioral experiments, we examined the consequences of constant photoinhibition of the BLA-EC glutamatergic pathway during the expression of contextual fear. Photoinhibition of the BLA-EC pathway did not alter conditioned contextual freezing in experimental mice compared to controls (repeated measures ANOVA NpHR3.0 vs. eYFP: *F*_1,15_ = 1.354; *P* = 0.263) (Figures 2B, F). These data support the hypothesis that activity of the BLA-EC glutamatergic pathway regulates acquisition but not expression of contextual fear.

## Discussion

Here, we show that the BLA sends a substantial glutamatergic projection to the EC, part of the hippocampal formation. Additionally, photoinhibition of this pathway during the acquisition, but not expression disrupted contextual fear conditioning. Our findings complement and extend previous work demonstrating a role of both the BLA and EC in contextual fear conditioning (Ueki et al., 1994; Maren and Fanselow, 1997; Majchrzak et al., 2006; Ji and Maren, 2008; Zovkic and Sweatt, 2013). Importantly, our data indicates this effect is modulated by direct inputs from the BLA to the EC.

Since our findings demonstrate a role for the BLA-EC pathway during contextual fear conditioning, our data are consistent with the idea that the hippocampal formation integrates contextual input with information about the US. This is in agreement with previous findings showing that the hippocampus functions as a temporary storage area of contextual fear conditioning (Anagnostaras et al., 1999). Although we were able to block the expression of contextual fear by photoinhibiting the glutamatergic projection from the BLA to the EC during acquisition, future studies should examine whether photostimulation of this pathway paired with a novel context can induce expression of fear.

We claim that our manipulations using *CamKIIα* as our promoter to drive ChR2 expression targets glutamatergic neurons. However, we did observe some expression in the central nucleus of the amygdala (CeA), a structure thought to be comprised primarily of GABAergic neurons. Interestingly, a recent study demonstrated that both GABA and glutamate neurons can be readily targeted with viral constructs using a *CamKIIα* promoter (Jennings et al., 2013). Since the BLA and its projections to the EC are glutamatergic and our slice electrophysiology data revealed that activation of ChR2-transduced terminals released glutamate, off target labeling of CeA likely did not affect the behavioral findings.

Our data suggests that that BLA-EC glutamatergic pathway mediates the acquisition, but not the expression of contextual fear. A candidate neural circuit involved in the mediation of the expression of this behavior may be the hippocampus to amygdala projection. Blockade of the hippocampus attenuates the renewal of conditioned fear (Maren and Hobin, 2007). Importantly, amygdala-projecting hippocampal neurons are active during the renewal of the fear response (Herry et al., 2008; Knapska et al., 2012). Additionally, the hippocampus can indirectly affect amygdala neurons through its projection to the medial prefrontal cortex (mPFC), which in turns projects to the amygdala. Disconnection of the hippocampus from the amygdala and PFC disrupts fear renewal, suggesting an integral role for this projection in the expression of contextual fear (Orsini et al., 2011). Thus, futures studies should examine whether optogenetic inhibition of the amygdala-projecting hippocampus and/or amygdala-projecting mPFC neurons can prevent the expression of contextual fear.

Although, our manipulations preferentially target the EC, we cannot rule out that our effects could be due to inhibition of the CA1 from the BLA. BLA glutamatergic neurons also project to the ventral hippocampus to modulate anxiety as well as social behavior in mice (Felix-Ortiz et al., 2013; Felix-Ortiz and Tye, 2014). Interestingly, inhibition of the BLA-ventral hippocampus pathway resulted in a decreases of anxiety-like behavior (Felix-Ortiz et al., 2013). In our findings, we observed decreases in freezing behavior in response to the fear context. It is possible that the decrease in freezing could be attributed to decrements in anxiety resulting from inhibition of the BLA-ventral hippocampus pathway. Given the close proximity and interconnectivity between the EC and CA1 it is extremely difficult to definitively parcel optogenetic effects on these two structures. However, we did observe a large percentage of EC neurons that received direct synaptic input from the BLA in our slice electrophysiology experiments (49/56 neurons), indicating a strong functional projection. This does not preclude the possible effects of inhibition of CA1 pyramidal neurons (via BLA fiber inhibition) as an additional circuit contribution. In conclusion, we provide evidence that the BLA-EC glutamatergic circuit encodes contexts associated with aversive or fearful stimuli and can be a potential pharmacological target for the treatment of anxiety disorders such as PTSD.

## Conflict of interest statement

The authors declare that the research was conducted in the absence of any commercial or financial relationships that could be construed as a potential conflict of interest.
